# Liver dysfunction is associated with long-term mortality in septic shock

**DOI:** 10.1186/cc14616

**Published:** 2015-03-16

**Authors:** N Nesseler, Y Launey, C Aninat, J White, P Seguin, Y Mallédant

**Affiliations:** 1 CHU Pontchaillou, Rennes, France; 2 Duke University Medical Centre, Durham, NC, USA

## Introduction

Knowledge of the impact of liver dysfunction on mortality during septic shock is limited. However, the liver appears to play a key role during septic illness. To better explore this issue, we investigated the data collected during the Prospective Recombinant Human Activated Protein C Worldwide Evaluation in Severe Sepsis and Septic Shock (PROWESS-SHOCK) trial in which a cohort of 1,697 septic shock patients were constituted [1]. The study aimed to assess the relationship of liver dysfunction at the onset and during the course of septic shock on short-term and long-term mortality.

## Methods

All of the patients enrolled in the PROWESS-SHOCK were included. Liver dysfunction at baseline was defined by a liver Sequential Organ Function Assessment (SOFA) score >0. The onset of a liver dysfunction post baseline was defined as any post-baseline increase of the hepatic SOFA score from 0. The worsening of liver dysfunction post baseline was defined as any increase of the hepatic SOFA score from the baseline assessment. The post-baseline period studied extended from study drug infusion to day 28. The main outcome was the mortality at day 180, and the secondary outcomes were the mortality at day 28 and at day 90. Cox proportional hazard models were used to estimate the hazard ratio of death.

## Results

Baseline liver function was assessed in 1,565 patients. Of those, 522 (33%) exhibited liver dysfunction at baseline. No relationship was found with mortality according to baseline liver dysfunction status at day 28 (HR, 0.847 (95% CI, 0.647 to 1.109); *P *= 0.2267), day 90 (HR, 0.883 (95% CI, 0.701 to 1.112); *P *= 0.2892) and day 180 (HR, 0.885 (95% CI, 0.710 to 1.103); *P *= 0.2761). A total of 403 (26%) patients developed a new liver dysfunction (257/1,043, 25%) or worsened liver dysfunction (146/522, 28%) in the 28-day post-baseline period. The overall median time to develop or to worsen liver dysfunction was 2 (1 to 4) days. Significant relationships between the new onset or the worsening of liver dysfunction post baseline and mortality at day 28 (HR, 1.67 (95% CI, 1.26 to 2.21); *P *= 0.0004), day 90 (HR, 1.65 (95% CI, 1.30 to 2.09); *P *< 0.0001) and day 180 (HR, 1.57 (95% CI, 1.26 to 1.97); *P *< 0.0001) were found.

## Conclusion

The new onset or the worsening of liver dysfunction during the course of septic shock impacts strongly on long-term mortality. Septic patients with liver dysfunction need long-term follow-up. Future research should focus on developing specific septic liver therapeutics and new tools for earlier detection of septic liver dysfunction.
